# Anti-GQ1b antibody syndrome presenting with visual deterioration as the initial symptom: A case report

**DOI:** 10.1097/MD.0000000000018805

**Published:** 2020-01-24

**Authors:** Teng Zhao, Yuyan Deng, Ying Ding, Rensheng Zhang, Chunkui Zhou, Weihong Lin

**Affiliations:** a Department of Neurology, The First Hospital of Jilin University, Changchun, Jinlin; b Department of Intensive Care Unit, the People's Hospital of Weifang City, Weifang, Shandong; c Department of Radiology, The First Hospital of Jilin University, Changchun, Jinlin, China.

**Keywords:** anti-GQ1b antibody syndrome, visual deterioration

## Abstract

**Rationale:**

Anti-GQ1b antibody syndrome refers to a distinct variant of Guillain– Barré syndrome. Involvement of the optic nerve in anti-GQ1b antibody syndrome is extremely rare.

**Patient concerns:**

Here, we report a case of anti-GQ1b antibody syndrome presenting with visual deterioration as the initial symptom. A 73-year-old man presented with a 5-day history of bilateral blurred vision and ptosis. He had a previous history of diarrhea starting 10 days before admission. Physical examination showed visual deterioration, ophthalmoplegia, and peripheral facial paralysis. Testing of both serum and cerebrospinal fluid was positive for anti-GQ1b immunoglobulin G antibodies and negative for anti-aquaporin 4antibodies.

**Diagnosis:**

Anti-GQ1b antibody syndrome.

**Interventions:**

The patient was treated with intravenous methylprednisolone and human immunoglobulin.

**Outcomes:**

After a 20-day follow-up, the patient's condition took a favorable turn.

**Lessons:**

This case reminds us that anti-GQ1b antibody syndrome should be suspected in patients with visual deterioration and preceding infection.

## 1 Introduction

Anti-GQ1b antibody syndrome refers to a distinct variant of Guillain–Barré syndrome that clinically includes Miller Fisher syndrome, Bickerstaff brainstem encephalitis, and acute ophthalmoplegia.^[1]^ Anti-GQ1b antibody is usually associated with impairments in motor cranial nerves, intrafusal type Ia afferent fibers, and dorsal root nerves.^[2]^ However, involvement of the optic nerve in anti-GQ1b antibody syndrome is extremely rare. Herein, we report a case of anti-GQ1b antibody syndrome presenting with visual deterioration as the initial symptom.

## 2 Case report

**Figure 1:**
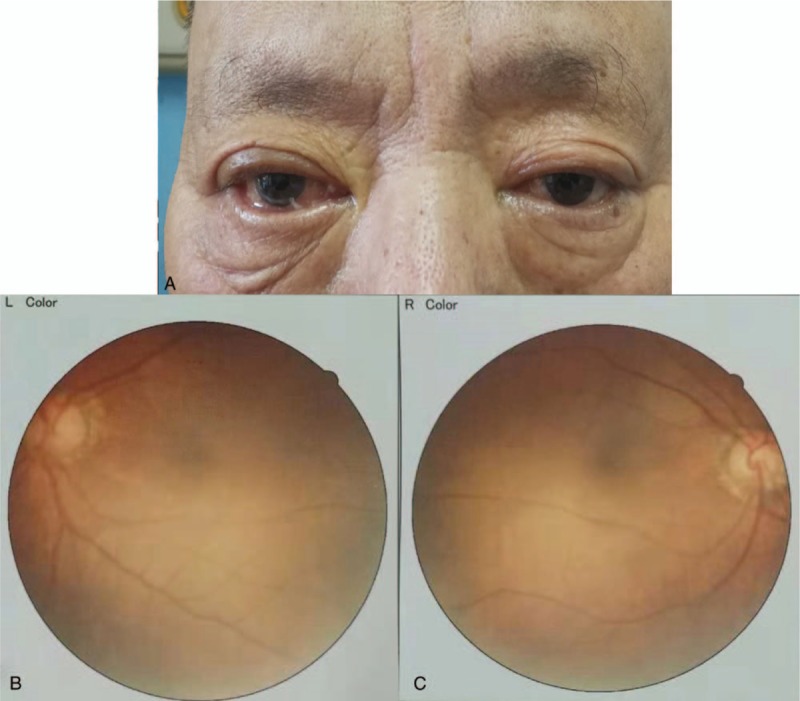
Facial and ocular features and fundus examination. (A) The patient's bilateral frontal and nasolabial grooves had become shallow, and the bilateral eyelash signs were positive. (B) The eyes were fixed with limited movement, and ptosis was noted; the pupil diameter in both eyes was 4 mm, and pupillary reaction to light was absent. (C and D) Fundus examination was normal.

**Figure 2:**
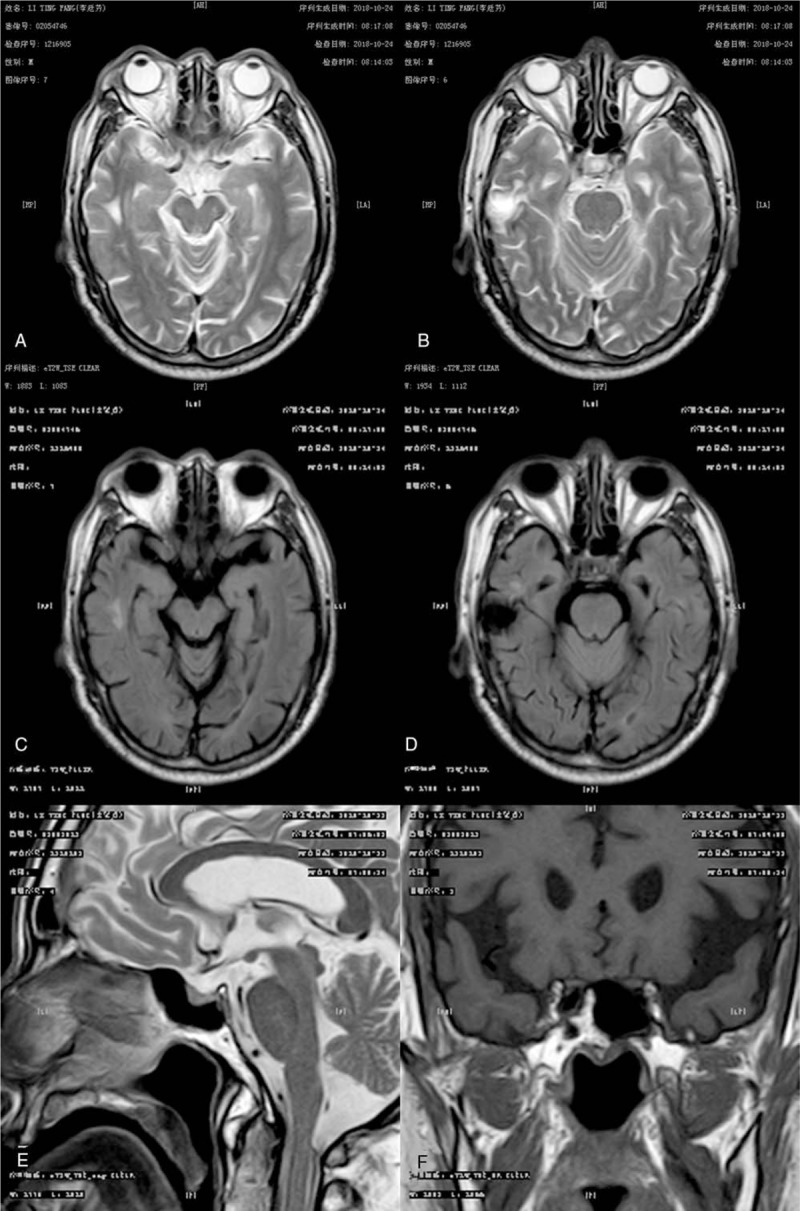
Brain magnetic resonance imaging. Brain magnetic resonance imaging showed no abnormality (A and B: axial T2-weighted imaging; C and D: axial T1-weighted imaging; E: sagittal T2-weighted imaging; F: coronal T1-weighted imaging).

A 73-year-old man presented to us with a 5-day history of bilateral blurred vision and ptosis. Ten days before admission, he suffered from acute diarrhea, and the feces was paste-like. Five days before admission, he developed severe blurred vision in both eyes; he could only perceive hand motion and could not recognize the number of fingers at a distance of 20 cm in front of the eyes. Additionally, his eyes were fixed, and bilateral ptosis was noted. Two days before admission, he developed weakness in eyelid closure, drooling at the mouth, and barylalia. There was no limb movement dysfunction, dysphagia, or choking. Forty years before, he had been diagnosed with ocular Guillain–Barré syndrome due to visual disturbance, which was cured by corticosteroid treatment. Physical examination showed elevated blood pressure (160/85 mm Hg), bilateral visual deterioration (right 0.6 and left 0.3), and dysarthria. The patient's bilateral frontal and nasolabial grooves were shallow, and the bilateral eyelash signs were positive. The eyes were fixed with limited movement, and ptosis was noted. The diameter of the pupil was 4 mm, and pupillary reaction to light was absent (Fig. 1A and B). Fundus examination was normal (Fig. 1C and D). The muscle strength and muscular tone were normal in all extremities, and there was no sensory disturbance or ataxia. Tendon hyporeflexia was observed in all limbs, and no pathological reflex was noted. Brain magnetic resonance imaging showed no abnormality (Fig. 2). Laboratory tests showed a glycosylated hemoglobin of 7.7% and a fasting blood-glucose level of 8.7 mmol/L. The results of cerebrospinal fluid examination were as follows: pressure 90 mmH_2_O, leukocyte count 2 × 10^6^/L, protein concentration 670 mg/L, glucose concentration 5.9 mmol/L, and chlorine concentration 126 mmol/L. Testing for anti-GQ1b immunoglobulin G antibodies was positive in both serum and cerebrospinal fluid, and that for anti-aquaporin 4 (AQP-4) antibodies was negative. A diagnosis of anti-GQ1b antibody syndrome was made. The patient was treated with intravenous methylprednisolone (500 mg/d) for 2 days, and his visual acuity was significantly improved (he could recognize the number of fingers at a 20-cm distance in front of the eyes). After the identification of anti-GQ1 b antibodies, human immunoglobulin (32.5 g/d) was administered for 5 days. After a 20-day follow-up, his visual acuity had recovered completely (right 0.9 and left 0.8). In addition, the facial paralysis was significantly relieved, and the eye movements were normal.

## 3 Discussion

Anti-GQ1b antibody syndrome is caused by microbial infection, such as *Campylobacter jejuni* and *Haemophilus influenzae*.^[3]^ These bacteria induce the production of anti-GQ1b antibodies in the central and peripheral nervous system; these antibodies bind to GQ1b antigens in the extramedullary part, neuromuscular junction, intrafusal type Ia afferent fibers, and dorsal root nerves of human brain nerves (III, IV, VI, VII, IX, and X), resulting in a spectrum of autoimmune diseases.^[4]^ In addition, anti-GQ1b antibodies are also highly expressed in the reticular activation system of the brain stem.^[5]^ Although anti-GQ1b antibodies can be detected in the optic nerve, previous reports of optic impairment in anti-GQ1b antibody syndrome have been limited due to the low incidence.^[6,7]^ Lamotte et al reported a case of anti-GQ1b antibody syndrome manifesting as eyelid retraction, visual loss, and ophthalmoplegia.^[1]^ Kauser et al reported a case of anti-GQ1b antibody syndrome presenting with bilateral ophthalmoplegia and unilateral facial paralysis.^[5]^ To our knowledge, the present case may be the first reported case with visual deterioration, ophthalmoplegia, and peripheral facial paralysis.

In the current case, the patient had a preceding history of infection and presented clinically with visual deterioration, ophthalmoplegia, and peripheral facial paralysis. Cerebrospinal fluid examination showed protein–cell separation and positivity for anti-GQ1b antibodies, and all of these findings were consistent with the diagnostic criteria of anti-GQ1b antibody syndrome.^[4]^ At present, the treatment of anti-GQ1b antibody syndrome has not been standardized, and most treatment modalities for Guillain–Barré syndrome are effective for anti-GQ1b antibody syndrome. The current case was sensitive to methylprednisolone and human immunoglobulin. Anti-GQ1b antibody syndrome is associated with a favorable prognosis and is generally not life-threatening. However, some cases may manifest as disturbance of consciousness, which may lead to severe pulmonary infection or other complications. The efficacy of immunoglobulin or plasma exchange is poor in these patients, and the risk of death should be of concern.^[8–10]^

## 4 Conclusion

This case reminds us that anti-GQ1b antibody syndrome should be suspected in patients with visual deterioration and preceding infection. A comprehensive serum and cerebrospinal fluid examination should be emphasized. The main differential diagnoses are neuromyelitis optica spectrum disorders, in which anti-AQP-4 antibodies are usually present and brain magnetic resonance imaging may show abnormal signals. The pathogenic mechanisms underlying the visual deterioration, ophthalmoplegia and peripheral facial paralysis require further research.

## Author contributions

**Software:** Ying Ding.

**Supervision:** Rensheng Zhang, Chunkui Zhou, Weihong Lin.

**Writing – original draft:** Yuyan Deng.

**Writing – review & editing:** Teng Zhao, Chunkui Zhou, Weihong Lin.
